# Drug users’ awareness of and willingness to use HIV non-occupational post-exposure prophylaxis (nPEP) services in China: a mixed methods study

**DOI:** 10.1186/s12879-022-07106-x

**Published:** 2022-02-14

**Authors:** Kedi Jiao, Haochu Li, Dapeng Zhang, Zhenxia Jiang, Yuxi Lin, Xueyuan Liu, Hengmin Xu, Xuemei Yan, Haoqing Tang, Wei Ma

**Affiliations:** 1 Department of Epidemiology, School of Public Health, Cheeloo College of Medicine, Shandong University, 44 West Wenhua Road, Jinan, Shandong 250012 People’s Republic of China; 2 Blue City, Suite 028, Building 2, North of Pinguo Neighborhood, 32 Baiziwan Road, Beijing, 100020 People’s Republic of China; 3 Department of HIV/STI Prevention and Control, Qingdao Municipal Center for Disease Control and Prevention, 175 Shandong Road, Qingdao, Shandong 266033 People’s Republic of China

**Keywords:** Drug user, HIV/AIDS, Non-occupational post-exposure prophylaxis, China

## Abstract

**Background:**

Drug users are regarded as a high-risk population for HIV infection. Non-occupational post-exposure prophylaxis (nPEP) is internationally regarded as an effective biomedical prevention against HIV but still a small-scale pilot project in China at present. The aim of this study was to understand drug users’ awareness of and willingness to use nPEP service in China.

**Methods:**

This mixed methods study consisting of a qualitative study and a cross-sectional survey was conducted in two cities of China from 2018 to 2019. The in-depth interviews were audio-taped, transcribed verbatim and analyzed using thematic framework analysis. Univariate and multivariate logistic regressions were used to examine factors associated with drug users’ awareness of and willingness to use nPEP.

**Results:**

There were 401 and 19 participants included in quantitative and qualitative study respectively. Among participants in quantitative study, 30.2% had heard of nPEP and 56.7% reported willingness to use nPEP in future HIV exposure. In multivariate analyses, nPEP awareness was associated with age, sex, education level, AIDS knowledge score and HIV risk perception. nPEP willingness was associated with AIDS knowledge score, HIV risk perception, alcohol use, monthly income and awareness of nPEP. The qualitative results showed the barriers to nPEP willingness included the fatigue after taking drugs, high cost and side effects of nPEP medication, long nPEP course, and fear of privacy disclosure.

**Conclusion:**

Drug users had low nPEP awareness and only about half participants reported willingness to use nPEP. It is essential to promote nPEP education campaigns among drug users, especially for elders, women and those with lower education level. Simultaneously, price regulation, side effect management, psychological support and privacy protection need to be managed well when nPEP is routinized.

**Supplementary Information:**

The online version contains supplementary material available at 10.1186/s12879-022-07106-x.

## Background

Illicit drug use is recognized as a contributor to the disease burden worldwide [1]. It was estimated that more than a quarter of billion people worldwide had used illicit drugs at least once in 2015 [2]. The number of newly HIV-infected injection drug users increased from 114,000 in 2011 to 152,000 in 2015, and the prevalence of HIV among injection drug users worldwide was 11.8% in 2016 [2]. Special attention should also be paid to the prevalence of club drugs that have been gradually increasing in many parts of the world over the past two decades [3]. The term “club drugs” refers to a category of drugs with a connection to clubs, “raves” or dance parties (e.g. methamphetamine, ketamine, Magu pills and ecstasy) [4]. In China, club drugs are commonly called new-type drugs for the reason that they are relatively new compared to “traditional drugs” (e.g. heroin, marijuana) [5]. In this study, the drug users were defined as people who used “traditional drugs” and/or the new-type drugs by any means including injection.

Post-exposure prophylaxis (PEP) is the use of antiretroviral drugs within 72 h of exposure to HIV in order to prevent infection [6]. PEP includes counseling, first aid care, HIV testing and administration of a 28-day course of antiretroviral drugs with follow-up care [6]. PEP was first used for HIV occupational exposure in the late 1980’s, and then gradually extended to non-occupational exposure including drug injection and sexual behavior [7]. The World Health Organization guidelines for PEP were first recommended in 2014 for all populations with both occupational and non-occupational exposures [8]. At present, non-occupational post-exposure prophylaxis (nPEP) service has been routinized in many regions of the world [9–11]. The guidelines of PEP in many countries (e.g. USA, UK and Belgian) have been issued for years and updated recently [12–14].

In China, needle sharing among injection drug users had declined dramatically in recent years with the efforts of national harm reduction programs, but the rapid emergence of new-type drugs brought a new challenge [15]. As of the end of 2019, there were about 2,148,000 drug users nationwide and 55.2% of them were methamphetamine abusers [16]. Studies have shown that the use of new-type drugs could increase the risk of HIV infection and transmission by encouraging high-risk sexual behaviors [17, 18]. There were 958,000 reported cases of people living with HIV/AIDS in China by 2019 [19]. The percentages of new HIV infection through drug injection decreased from 13% in 2012 to 2.4% in the third quarter of 2018, while sexual transmission increased from 79.5% in 2012 to 93.8% in the third quarter of 2018 [20, 21]. PEP has been widely used for occupational protection against HIV, while nPEP is just piloted in a small scale in China. Up to date, only a few studies focused on nPEP in China. A study in Guangxi Province reported that among 344 men who have sex with men (MSM), 22.1% had heard of nPEP and 60.2% reported willingness to use nPEP [22]. Another study in Jinan surveyed 171 men receiving nPEP and found no HIV conversion after nPEP [23].

Even fewer studies focused on the subgroup of drug users to explore their views about nPEP before the routinization of this service. The aim of this study was therefore to understand drug users’ awareness of and willingness to use nPEP service and explore potential barriers to nPEP willingness and suggestions for this service, which will facilitate the development of nPEP guideline and routinization of this service among drug users in China and worldwide.

## Methods

### Study design

The mixed methods study was conducted from August 2018 to March 2019 in Qingdao and Shanghai, two major coastal cities of China where illicit drug trafficking and abusing were common [5, 24]. Qualitative study was designed to guide the improvement of quantitative questionnaire and then utilized to explain and complement the quantitative results. This mixed approach not only assessed the awareness of and willingness to use nPEP from the perspective of participants but also identified the barriers to nPEP willingness that could be intervened upon.

### Participants

Convenience sampling was used to recruit participants through referral from community-based organizations (CBOs) and Centers for Disease Control and Prevention (CDC) in Qingdao and Shanghai. In-depth interviews were arranged in a private room in the local CDC or CBO. The participants were assured of confidentiality, privacy protection (e.g. use of pseudonyms), and their rights to refuse to answer any questions or withdraw at any stage without prejudice.

Eligible participants should: (1) reach 18 years old or above; (2) take illicit drugs including new-type drugs in the past 12 months; (3) be HIV-negative or unknown of HIV status; and (4) provide written informed consent and voluntarily participate in the survey. For the qualitative study, the participants should also live in the study sites for at least 3 months and were sampled with a wide range of age, occupation and education with the aim of capturing varieties of views within this population.

The eligible participants were compensated 200 Chinese Yuan (CNY) (about 29.24 USD) after completing the in-depth interview and 50 CNY (about 7.31 USD) after completing the structured questionnaire respectively for their time spent.

### Data collection

#### Qualitative data collection

The in-depth interviews were conducted by two trained interviewers using mandarin Chinese after the written informed consents were provided. Each interview lasted approximately 40–50 min. A semi-structured interview guide was developed according to a review of the literature, and collected information including drug use behaviors, high-risk sexual behaviors, nPEP awareness and usage, willingness to use nPEP in future exposure and reasons, potential impacts of nPEP routinization, and suggestions for nPEP implementation in future.

#### Quantitative data collection

After providing written informed consents, the participants were required to complete a structured questionnaire (either in an online version via Wenjuanxing or a paper version). We developed the questionnaire by referring to China's HIV/AIDS sentinel surveillance questionnaire and adapted it based on literature review and qualitative study (see Additional file 1). The questionnaire covered the following variables: age, sex, local household, education level, monthly income, marital status, AIDS knowledge score, utilization of HIV prevention services, alcohol use, drug use, high-risk sexual behavior, and HIV risk perception. The outcome variables included awareness of and willingness to use nPEP (see Additional file 2 for details).

### Data analysis

#### Qualitative data analysis

All interviews were audio-taped, transcribed verbatim in Chinese and analyzed using thematic framework analysis [25]. First, the authors read the transcripts carefully to gain a comprehensive understanding to list key ideas and recurrent themes. Next, a thematic framework (including nPEP awareness, willingness and suggestions) was developed and applied to code each transcript using ATLAS.ti 5.0. By inductive reasoning, we identified participants’ awareness of nPEP, barriers of willingness to use nPEP, and suggestions for nPEP (including endorsement of routinization, preferred institutions providing nPEP and affordable nPEP price). Accompanying quotes and related themes were then clustered and synthesized. Finally, the first author translated the relevant quotes into English and the second author made cross-check.

#### Quantitative data analysis

Frequencies were used to describe demographic and behavioral characteristics. Univariate and multivariate logistic regressions were used to examine factors associated with the two outcomes (i.e. awareness of nPEP and willingness to use nPEP). Variables with P ≤ 0.10 in the univariate analyses were included in the multivariate logistic regression models. In multivariate analyses, a forward likelihood ratio method was used to select variables. Adjusted odds ratios (AOR) with 95% confidence intervals (CI) and P-values were reported. All statistical analyses and plots were conducted using SPSS 24.0 and R 4.0.1 respectively with two-side P < 0.05 taken as statistically significant.

### Ethical approval

The study was approved by the Ethical Review Committee of School of Public Health in Shandong University (20180904).

## Results

### Quantitative results

#### Sample characteristics

For quantitative study, a total of 401 eligible participants were included in the analysis. Among them, about 40% were 30 years old and below, 59.5% were male, 73.8% were local household, 59.3% had an education level of senior high school and below, 47.1% earned a monthly income at or above 3000 CNY, and 75.6% were currently unmarried. Nearly 60% and 65% participants used illicit drugs and alcohol respectively in the past 3 months, 35.4% participants had condomless sex after using drugs in the past year. There were 30.2% and 56.7% participants having heard of nPEP and reporting willingness to use nPEP in future HIV exposure respectively (Table 1).

**Table 1:** Demographic and behavioral characteristics of drug users in two cities of China (N = 401)

Variables	Total sample ^a^	Having heard of nPEP			Willing to use nPEP		
	N = 401 (%)	Yes, n = 120 (%)	No, n = 277 (%)	χ^2^	Yes, n = 216 (%)	No, n = 165 (%)	χ^2^
Age (years)				49.094^***^			23.092^***^
≤ 30	158 (39.6)	79 (65.8)	79 (28.7)		110 (51.2)	47 (28.7)	
31–40	96 (24.1)	20 (16.7)	74 (26.9)		50 (23.2)	41 (25.0)	
> 40	145 (36.3)	21 (17.5)	122 (44.4)		55 (25.6)	76 (46.3)	
Sex				41.013^***^			5.887^*^
Male	237 (59.5)	100 (83.3)	134 (48.9)		140 (65.1)	86 (52.8)	
Female	161 (40.5)	20 (16.7)	140 (51.1)		75 (34.9)	77 (47.2)	
Local household				22.779^***^			7.048^**^
Yes	296 (73.8)	69 (57.5)	223 (80.5)		145 (67.1)	131 (79.4)	
No	105 (26.2)	51 (42.5)	54 (19.5)		71 (32.9)	34 (20.6)	
Education level				37.006^***^			5.363^*^
Senior high school and below	229 (59.3)	43 (36.1)	182 (69.2)		112 (52.8)	100 (64.9)	
College and above	157 (40.7)	76 (63.9)	81 (30.8)		100 (47.2)	54 (35.1)	
Monthly income (CNY)				3.258			11.152^**^
< 1500	78 (19.7)	20 (17.2)	56 (20.4)		30 (14.1)	43 (26.4)	
1500–3000	131 (33.2)	33 (28.5)	97 (35.3)		80 (37.5)	42 (25.8)	
> 3000	186 (47.1)	63 (54.3)	122 (44.4)		103 (48.4)	78 (47.8)	
Marital status				0.022			< 0.001
Currently unmarried	297 (75.6)	89 (76.1)	205 (75.4)		161 (75.6)	121 (75.6)	
Currently married	96 (24.4)	28 (23.9)	67 (24.6)		52 (24.4)	39 (24.4)	
AIDS knowledge score				22.213^***^			58.672^***^
0–5	161 (40.6)	27 (22.9)	133 (48.4)		51 (23.6)	101 (62.7)	
6–8	236 (59.4)	91 (77.1)	142 (51.6)		165 (76.4)	60 (37.3)	
Utilization of HIV prevention services in the past year				0.245			2.779
0–1	95 (23.9)	26 (22.2)	68 (24.5)		45 (20.8)	46 (28.2)	
2–3	303 (76.1)	91 (77.8)	209 (75.5)		171 (79.2)	117 (71.8)	
Ever use multiple drugs				1.157			0.006
Yes	114 (28.5)	39 (32.5)	75 (27.2)		62 (28.8)	47 (28.5)	
No	286 (71.5)	81 (67.5)	201 (72.8)		153 (71.2)	118 (71.5)	
Drug use in the past 3 months				21.988^***^			9.156*
Never	152 (38.9)	28 (23.9)	120 (44.4)		81 (38.6)	54 (32.9)	
Occasionally	120 (30.7)	35 (29.9)	85 (31.5)		54 (25.7)	66 (40.3)	
More than once a month	119 (30.4)	54 (46.2)	65 (24.1)		75 (35.7)	44 (26.8)	
Condomless sex after using drugs in the past year				17.546^***^			9.069^**^
Yes	142 (35.4)	61 (51.3)	81 (29.2)		94 (43.5)	47 (28.5)	
No	257 (64.6)	58 (48.7)	196 (70.8)		122 (56.5)	118 (71.5)	
Condomless group sex after using drugs in the past year				7.405^**^			8.344^**^
Yes	55 (13.8)	25 (21.2)	30 (10.8)		41 (19.0)	14 (8.5)	
No	343 (86.2)	93 (78.8)	247 (89.2)		175 (81.0)	151 (91.5)	
Alcohol use in the past 3 months				3.388			29.328^***^
Yes	262 (65.8)	71 (59.7)	191 (69.2)		121 (56.0)	135 (82.3)	
No	136 (34.2)	48 (40.3)	85 (30.8)		95 (44.0)	29 (17.7)	
HIV risk perception				38.703^***^			46.866^***^
Not serious or have no idea	215 (53.6)	39 (32.5)	175 (63.2)		99 (45.8)	103 (62.4)	
Moderate	116 (28.9)	58 (48.3)	55 (19.8)		92 (42.6)	19 (11.5)	
Serious	70 (17.5)	23 (19.2)	47 (17.0)		25 (11.6)	43 (26.1)	
Having heard of nPEP				NA			45.059^***^
Yes	120 (30.2)	NA	NA		95 (44.0)	20 (12.1)	
No	277 (69.8)	NA	NA		121 (56.0)	145 (87.9)	

nPEP, non-occupational post-exposure prophylaxis; CNY, Chinese Yuan (1 CNY = 0.14 USD); NA, not appliacable

^a^ The total sample size was 401. Due to participant choice to refrain from answering some questions, denominators can vary from 401

^*^ P < 0.05

^**^ P < 0.01

^***^ P < 0.001

The results of univariate analyses targeting the two outcome variables were displayed in Additional file 3: Table S1.

#### Factors associated with awareness of nPEP service

**Fig. 1:**
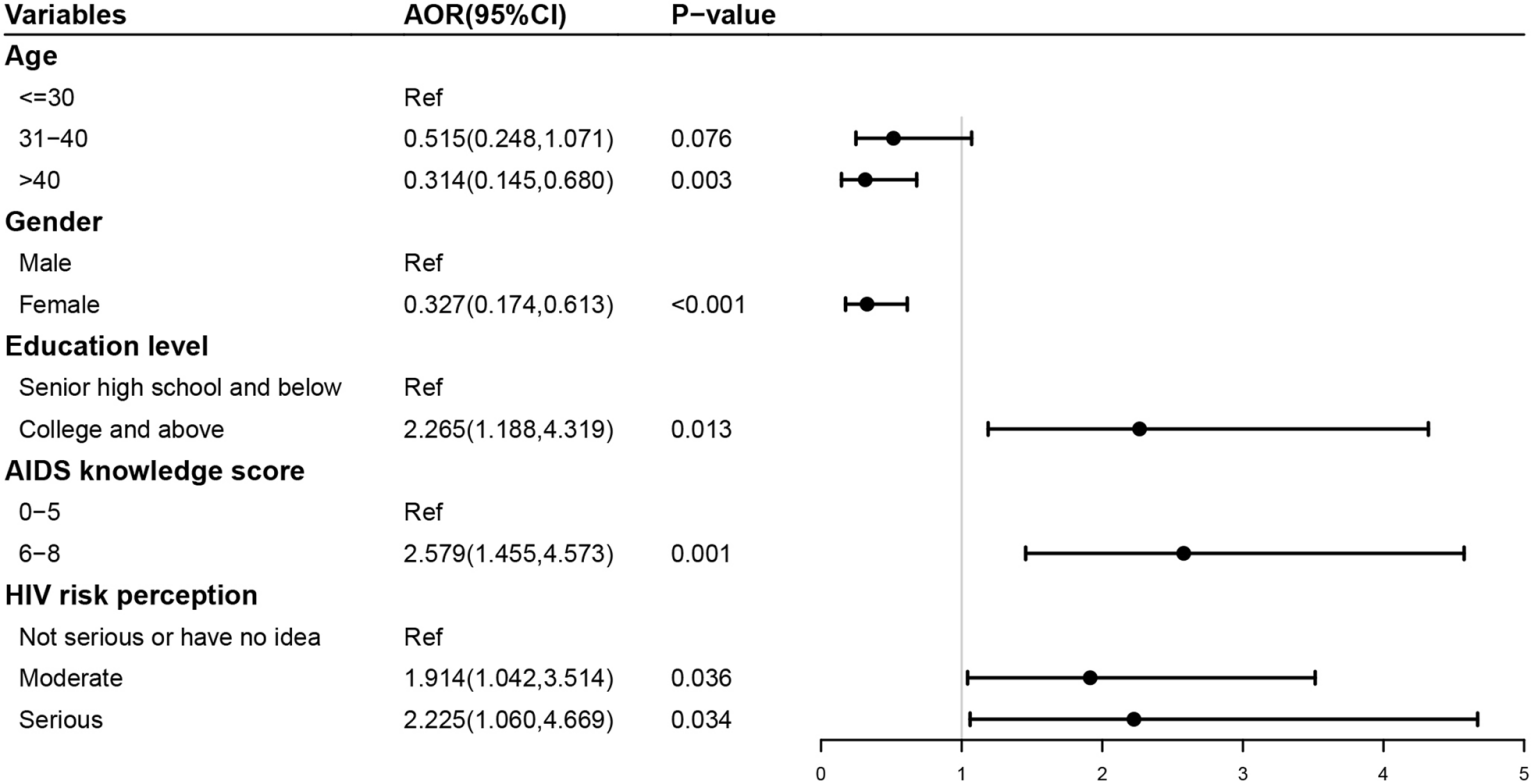
Multivariate logistic regression of factors associated with awareness of nPEP among drug users in China. AOR, adjusted odds ratio; CI, confidence interval; nPEP, non-occupational post-exposure prophylaxis

As shown in Fig. 1, the likelihood of awareness of nPEP was lower among those aged > 40 years old (AOR = 0.314, 95%CI: 0.145–0.680) and women (AOR = 0.327, 95%CI: 0.174–0.613). Conversely, higher education level (AOR = 2.265, 95%CI: 1.188–4.319), higher AIDS knowledge score (AOR = 2.579, 95%CI: 1.455–4.573), and higher HIV risk perception (AOR = 1.914, 95%CI: 1.042–3.514; AOR = 2.225, 95%CI: 1.060–4.669) were positively associated with nPEP awareness.

#### Factors associated with willingness to use nPEP service

**Fig. 2:**
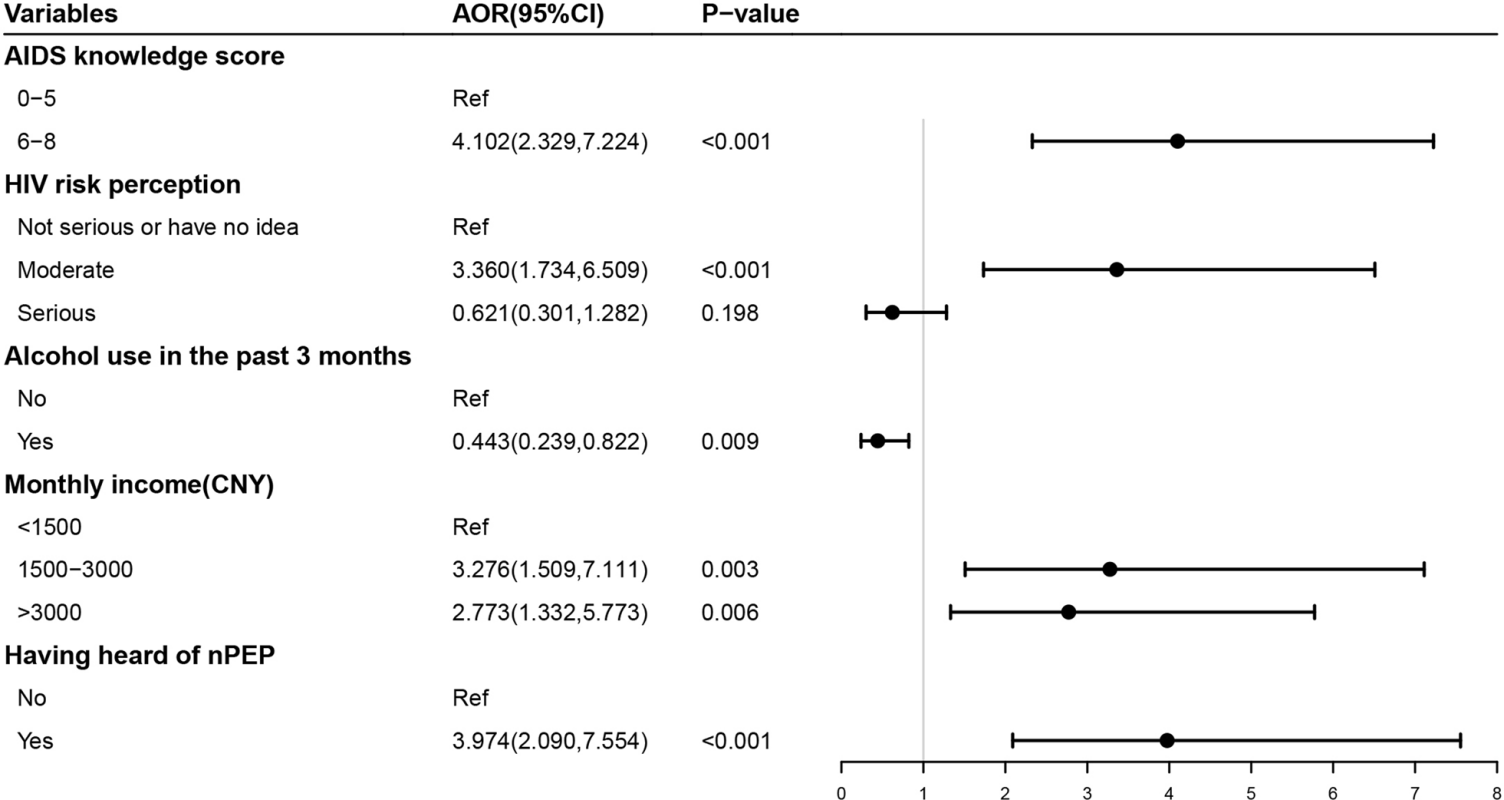
Multivariate logistic regression of factors associated with willingness to nPEP among drug users in China. AOR, adjusted odds ratio; CI, confidence interval; CNY, Chinese Yuan (1 CNY = 0.14 USD); nPEP, non-occupational post-exposure prophylaxis

Those having higher AIDS knowledge score (AOR = 4.102, 95%CI: 2.329–7.224), perceiving moderate risk of HIV infection among drug users in their living city (AOR = 3.360, 95%CI: 1.734–6.509), having higher monthly income (AOR = 3.276, 95%CI: 1.509–7.111; AOR = 2.773, 95%CI = 1.332–5.773) and having heard of nPEP service (AOR = 3.974, 95%CI: 2.090–7.554) were more likely to report willingness to use nPEP. While, those who used alcohol in the past 3 months (AOR = 0.443, 95%CI: 0.239–0.822) were less likely to report willingness to use nPEP (Fig. 2).

#### Sex-specific analysis

For men, nPEP awareness was significantly associated with younger age, higher AIDS knowledge score and perceiving moderate risk of HIV infection among drug users. Willingness to use nPEP was significantly associated with higher monthly income, higher AIDS knowledge score, perceiving moderate risk of HIV infection among drug users, utilization of HIV prevention services and having heard of nPEP. While for women, nPEP awareness was significantly associated with higher education level and no alcohol use in the past 3 months. Willingness to use nPEP was significantly associated with no local household, higher AIDS knowledge score, having condomless sex after using drugs in the past year and no alcohol use in the past 3 months (see details in Additional file 3: Tables S2–S7).

### Qualitative results

#### Sample characteristics

A total of 19 eligible participants were recruited in the qualitative study, including 10 in Shanghai and 9 in Qingdao. Among the participants, 42.1% were aged 30 and below, 73.7% were male and 84.2% were employed at present. About 60% participants earned a monthly income below 5000 CNY. Most of participants (63.2%) were never married (Table 2).

**Table 2:** Socio-demographic characteristics of in-depth interview participants (N = 19)

Characteristics	Number (%)
Age (years)	
≤ 30	8 (42.1)
31–49	6 (31.6)
≥ 50	5 (26.3)
Sex	
Male	14 (73.7)
Female	5 (26.3)
Education level	
High school and below	9 (47.4)
College and above	10 (52.6)
Occupation	
Employed	16 (84.2)
Unemployed	3 (15.8)
Monthly income (CNY)	
< 5000	11 (57.9)
≥ 5000	8 (42.1)
Marital status	
Married	4 (21.0)
Divorced	3 (15.8)
Never married	12 (63.2)

CNY, Chinese Yuan (1 CNY = 0,14 USD)

#### Awareness of nPEP service

Most (17/19) participants reported having never heard of nPEP. Only two participants had heard of nPEP through Internet and communications with the salesman in a pharmacy respectively. However, neither of them had clear knowledge about nPEP or how to get access to nPEP services.

*I glanced at it (nPEP) on the website but without further understanding the service. (33-year-old, SH01)*

#### Willingness to use nPEP service and potential barriers

After being introduced about the basic knowledge of nPEP, only 36.8% of the participants reported willingness to use this service in future exposure and five barriers were identified. First, there were participants claiming that it was difficult for them to seek nPEP within recommended 72 h due to fatigue and lethargy after taking drugs.

*Someone just like me will choose to sleep for a day or one and a half days after taking drugs. Some others with violent temper are impossible to keep calm. The staying power of drugs is too strong for me to keep in mind in taking medications (nPEP). (24-year-old, QD05).*

Second, some participants reported that they could not afford nPEP services, especially for those who had lower income. Nevertheless, some other participants claimed that they would use nPEP at any cost if exposed to HIV.

*The biggest barrier (to use nPEP) is the high price which are unaffordable for most people. Only those with high income can afford it. (57-year-old, SH04)*

*I can accept the price. I have to weigh the pros and cons. What should I do if I suffer from AIDS? I may lose my job. I just spend money to protect myself. (27-year-old, QD09)*

Third, side effects of nPEP medications might be another concern. Some participants argued that they were not willing to use nPEP if there were some particular side effects.

*I worry about the side effects. I spend a lot of money improving my appearance. As a result, it’s desirable to have no side effects such as alopecia, skin rash and so on. (23-year-old, QD06)*

*I worry about the side effects of the medicine because of my allergic history. I am allergic to something and fear that it interacts with the nPEP medicines. (24-year-old, QD01)*

Fourth, some participants indicated that a 28-day course of nPEP was too long for them to keep compliance. For one thing, they had difficulties in persistently taking medications every day. For another, some participants thought that some peers in their circles may have unprotected sex again within a nPEP course.

*It may be feasible to explain to friends that I catch a cold but impossible to maintain this condition for 28 days. The course of nPEP medication is so long that my colleagues may suspect. (24-year-old, QD05)*

*I suppose that they (other drug users) definitely (have sex) more than once in a month. However, they have pretty high likelihood to have sex again during a nPEP course. (24-year-old, QD01)*

Fifth, fear of privacy disclosure when seeking nPEP in the hospital was another barrier to willingness to use this service.

*This kind of thing (suffering from AIDS) is disgraceful and confidential. For example, I will go to the hospital for checkup after high-risk sexual behavior. If there are a lot of patients around, I will feel humiliated if the doctors don’t hear clearly and asked me “what” loudly. If I am HIV-positive and the doctors consistently repeat, I will feel embarrassed. In this regard, a private institution rather than hospital is more suitable for providing nPEP. (24-year-old, QD05).*

Besides, the participants gave some suggestions on endorsement of nPEP routinization, preferred institutions providing nPEP and affordable price. Most participants supported the routinization of nPEP among drug users and chose social media for nPEP propaganda and pharmacies for providing nPEP respectively. The current price of a course of nPEP was assumed to be high and advised to set at 1000–2000 CNY (see details in Additional file 4).

## Discussion

This study used a mixed method to understand drug users’ awareness of and willingness to use nPEP services in China. Both qualitative and quantitative studies reported low nPEP awareness among drug users. Demographic and behavioral factors influencing awareness of and willingness to use nPEP were examined through quantitative study. Meanwhile, potential barriers to nPEP willingness and suggestions for nPEP were explored through qualitative study.

Only 2 (10.5%) participants in qualitative study and 120 (30.2%) participants in quantitative study had heard of nPEP before this study. Similarly, a Chinese study targeting MSM conducted in 2017 found that only 22.1% of participants had heard of nPEP [22]. This may be due to that nPEP services were just piloted in a small scale and had not been included into routine HIV/AIDS campaigns in China yet. There were 56.7% participants in quantitative study reported willingness to use nPEP in the future HIV exposure, which was similar with another study (60.2%) in China [22] but lower than a study (73%) in U.S. [26]. This finding indicated that it is essential to strengthen nPEP education and promotion campaigns among key populations in China.

According to quantitative findings, elders (> 40 years old), especially for men, were less likely to have heard of nPEP than the younger ones (≤ 30 years old), which may be because they were less adaptive to access new HIV-related information compared with youngsters [27]. Female participants reported lower likeliness of nPEP awareness than their male counterparts. This may be due to that women, as vulnerable groups, were not easy to access HIV-related resources [28, 29]. Those with higher education level, higher AIDS knowledge score and higher risk perception were more likely to have heard of nPEP, which were consistent with previous studies [24, 30]. Therefore, nPEP education campaigns need to be tailored for drug users with specific characteristics, namely those aged > 40 years old, women and those with lower education level.

As for nPEP willingness, it was noteworthy that participants perceiving moderate risk of HIV infection among drug users in their living city instead of those perceiving serious HIV risk were more willing to use nPEP. The reason might be that those perceiving serious risk of HIV infection had higher self-protection perception and were more likely to use condom consistently, which may decrease the risk of exposure. Alcohol use was reported negatively associated with willingness to use nPEP. Evidence showed that alcohol can directly act upon the brain, resulting in the diminished risk perception and decrease the utilization of HIV-related prevention services [31, 32]. Few HIV policies or projects regarded alcohol use as a barrier of HIV-related services (e.g. nPEP). This study highlighted the significance of alcohol screening for drug users with alcohol problem (i.e. concurrent alcohol and drug users). It is necessary to integrate alcohol risk reduction into HIV prevention services [33]. Further sex-specific analysis showed female drug users who use alcohol were related with lower odds of nPEP awareness and willingness. Women may experience metabolic vulnerabilities and therefore they suffer negative consequences of alcohol use faster and to a greater degree than men [34]. Moreover, poorer people had lower odds of using nPEP in future HIV exposure. The high cost may be a key barrier for them to access nPEP and this is also corroborated by the qualitative study, which will be discussed in the following sections. Those having heard of nPEP were more likely to use this service, which indicated that HIV education campaigns may play an important role in nPEP promotion. Evidence has shown that nPEP education campaigns could significantly increase awareness of and willingness to use nPEP [35].

In the qualitative study, we identify five barriers to willingness to use nPEP services, including fatigue and lethargy after taking drugs, high price, side effects of nPEP medications, long nPEP course, and fear of privacy disclosure. The feeling of fatigue and lethargy would last for a few days (i.e. fatigue period) after taking drugs, which may prevent drug users to seek nPEP in time [36]. Increasing the coverage of nPEP services and improving convenience to access nPEP may play an important role, especially in high-risk areas. Besides, another option of pre-exposure prophylaxis (PrEP) could be planned and implemented. Different from nPEP, PrEP is the use of antiretroviral drugs before HIV-related high-risk behaviors in order to prevent infection. The high cost was also a barrier for accessing nPEP. Different from this study, a study in U.S. revealed that men with lower annual individual income were more likely to use nPEP [26]. But it was noteworthy that health insurance could often cover the costs of nPEP in U.S. and the study also suggested that those with lower incomes might have difficulty in paying for nPEP without financial support. Policymakers should notice that the affordable price of nPEP services for most participants should be no more than 2000 CNY and they should take the income levels in different areas into account when drafting the price of nPEP (see details in Additional file 4). It may be feasible to include nPEP medications into medical insurance in future. In Switzerland, the cost of nPEP is charged directly to patients and then partially reimbursed through medical insurance [10]. In previous studies, side effects were also one of the main barriers to using nPEP [37, 38]. But in general, most common side effects could be improved through symptomatic treatment during the follow up. An initial prescription for 3–5 days medication with a follow-up visit could be alternatively chose to assess medication side effects and provide additional counseling [39]. Moreover, some participants complained that a 28-day nPEP course was too long and inconvenient to take medicine every day. In this regard, Kahn et al. [40] gave suggestions including medication adherence education, psychological support and lowering pill burden. Besides, participants worried about the risk of privacy disclosure when seeking nPEP in the hospital, which may bring about stigma and discrimination. Previous study in China also suggested that HIV-related discrimination from healthcare providers could hinder the utilization of HIV prevention and treatment [41]. This indicated that a key priority was to strengthen privacy protection in the institutions providing nPEP.

There are several limitations in this study. First of all, a relatively small sample of drug users was included in this study and they were recruited using convenience sampling, which may limit the generalization of the findings. Second, self-reported data including sensitive information may lead to “information bias”. Third, causal relationship in quantitative study cannot be established due to the limitation of cross-sectional design.

## Conclusions

Drug user participants had low nPEP awareness, especially for elders (> 40 years old), women and those with lower education level. About half participants reported willingness to use nPEP, mainly including those with higher AIDS knowledge score, higher income and having heard of nPEP. It is essential to promote nPEP education campaigns among drug users, particularly key subgroups. Simultaneously, price regulation, side effect management, psychological support and privacy protection need to be managed well when nPEP is routinized.

## Supplementary Information

**Additional file 1.** Survey questionnaire.

**Additional file 2.** Variables and measures in quantitative study.

**Additional file 3.** Univariate analyses of the two outcome variables and sex-specific analyses.

**Additional file 4.** Suggestions for nPEP service in qualitative study.

## Data Availability

The datasets used and/or analysed during the current study are available from the corresponding author (Prof. Wei Ma, weima@sdu.edu.cn).

## Abbreviations

AOR: Adjusted odds ratio
CBO: Community-based organizations
CDC: Centers for Disease Control and Prevention
CI: Confidence interval
CNY: Chinese Yuan
MSM: Men who have sex with men
nPEP: Non-occupational post-exposure prophylaxis
PEP: Post-exposure prophylaxis
