# Supplementary material for: Drug users’ awareness of and willingness to use HIV non-occupational post-exposure prophylaxis (nPEP) services in China: a mixed methods study
Source: BMC Infect Dis. 2022 Feb 14;22:151. doi: 10.1186/s12879-022-07106-x (PMC8842954; doi:10.1186/s12879-022-07106-x)
Supplement: Supplementary file 1 — Additional file 1. Survey questionnaire. [file 12879_2022_7106_MOESM1_ESM.docx]

**Additional file 1 Survey questionnaire**

**Part 1. Basic information**

A01. What is your year of birth? _______

A02. What is your sex?

[1] Male [2] Female

A03. Where are you from?

[1] Qingdao [2] Shanghai

A04. What is your ethnicity?

[1] Han [2] Other ethnics:_______

A05. Where is your household registry?

[1] Local city [2] Other city in local province [3] Other province [_____Province] [4] Foreign nationality [____Country]

A06. How long have you lived here?

[1] <3 months [2] 3-6 months [3] 6-12 months [4] 1-2 years [5] >2 years

A07. What is your highest education level?

[1] Illiteracy [2] Primary school [3] Junior high school [4] Senior high school [5] College and above

A08. What is your employment?

[1] Leaders of enterprise [2] Professional technicians [3] Clerical and related personnel [4] Business and services personnel [5] Production personnel in agriculture, forestry, animal husbandry, fishery and water conservancy [6] Production and transportation equipment operators and relevant personnel [7] Soldier [8] Student [9] Other employees

A09. How much can you earn per month? (CNY=Chinese Yuan)

[1]<1500 CNY [2] 1500-3000 CNY [3] 3001-5000 CNY [4] 5001-8000 CNY [5] >8000 CNY

A10. What is your current marital status?

[1] Unmarried [2] Married [3] Cohabitating [4] Divorced/widowed/separated

**Part 2 AIDS knowledge and utilization of HIV prevention services**

B01. Is AIDS an incurable serious infectious disease?

[1] Yes [2] No [3] Not sure

B02. Is it possible to be infected with HIV if sharing syringes with PLWH？

[1] Yes [2] No [3] Not sure

B03. Could you judge if a person is infected with HIV by appearance？

[1] Yes [2] No [3] Not sure

B04. Could correct and consistent use of condoms reduce the risk of HIV acquisition and transmission？

[1] Yes [2] No [3] Not sure

B05. Could use of new-type illicit drugs (e.g., methamphetamine, ecstasy) increase the risk of HIV acquisition?

[1] Yes [2] No [3] Not sure

B06. Could drug maintenance therapy reduce the risk of HIV acquisition?

[1] Yes [2] No [3] Not sure

B07. Should you actively seek for HIV testing and consultation after high risk behaviors (e.g., sharing needle or unsafe sex)?

[1] Yes [2] No [3] Not sure

B08. Do you need to bear legal responsibility if you transmitted HIV intentionally?

[1] Yes [2] No [3] Not sure

B09. Did you receive any of the following HIV prevention services in the past year?

B09a. Condom promotion and distribution/ HIV counseling and testing services?

[1] Yes [2] No

B09b. Community-based methadone maintenance treatment/ needle exchange program services?

[1] Yes [2] No

B09c. Peer education services?

[1] Yes [2] No

**Part 3 Behavioral information**

C01. Which of the following drugs have you ever used? [Multiple choices]

[1] Heroin [2] Cocaine [3] Methamphetamine [4] Magu [5] Ketamine [6] Opium [7] Mariguana [8] Morphine [9] Sauteralgyl [10] Rush [11] Ecstasy [12] Other drugs:___________

C02.Which of the following ways have you used to take drugs? [Multiple choices]

[1] Inhaling [2] Oral [3] Snuff [4] Mixed with cigarettes [5] Injection [6] Other ways:

C03. Have you ever injected drugs in the past month? [1] Yes [2] No (Jump to C07)

C04. How many times did you inject drugs per day in the past month?

C05. Have you shared needles with others when you injected drugs in the past month?

[1] Yes [2] No (Jump to C07)

C06. How often did you share needles with others when you injected drugs in the past month?

[1] Sometimes (<50%) [2] Often (≥50%) [3] Always (100%)

C07. How many people do you usually use drugs with in the past year?

[1] Alone [2] 1-2 [3] 3-5 [4] 6-10 [5] >10

C08. How often did you use drugs on average in the past three months?

[1] Never [2] Occasionally [3] 1-3 times per month [4] 1-4 times per week [5] 5-7 times per week [6] More than 7 times per week

C09. Have you ever had sex after using drugs in the past year? [1] Yes [2] No (Jump to C15)

C10. Are your sexual partners usually homosexual or heterosexual after using drugs?

[1] Homosexual [2] Heterosexual [3] Both

C11. How often did you use condoms when you had sex after using drugs?

[1] Never use [2] Sometimes use [3] Always use

C12. Whether the condom has been broken, slipped or deliberately pulled out during the intercourse after using drugs?

[1] Yes [2] No [3] Unclear

C13. Have you ever had group sex after using drugs in the past year?

[1] Yes [2] No (Jump to C15)

C14. How often did you use condoms when you had group sex after using drugs?

[1] Never use [2] Sometimes use [3] Always use

C15. How often did you drink in the past three months?

[1] Never [2] Occasionally [3] 1-3 times per month [4] 1-4 times per week [5] 5-7 times per week [6] More than 7 times per week

C16. Have you been diagnosed with sexually transmitted diseases in the past 12 months?

[1] Yes [2] No

C17. How often did you participate in HIV testing?

[1] Never [2] Did not test for HIV in the past 12 months

[3] Once in the past 12 months [4] Twice or more in the past 12 months

**Part 4 Awareness of and willingness to use nPEP**

D01. What do you think of the risk of HIV infection among drug users in your living city?

[1] Not serious [2] Moderate [3] Serious [4] Have no idea

D02. Have you heard of nPEP services before taking part in this study?

[1] Yes [2] No (Jump to D06)

D03.Where did you hear of nPEP [Multiple choices]

[1] Friends [2] CBO [3] Hospital [4] CDC [5] Internet/Social media/APP

[6] Broadcast/TV/display broad [7] Others:____________

D04. Please judge whether the following statements are correct?

D04a. NPEP can be taken within one week after the occurrence of behaviours that may lead to the infection of HIV (e.g., unprotected sex, sharing needles).

[1] True [2] False [3] Unknown

D04b. A person does not need to take nPEP after having unprotected sex with people with unknown HIV status.

[1] True [2] False [3] Unknown

D04c. A person needs to take PEP after sharing a needle with people living with HIV or with unknown HIV status.

[1] True [2] False [3] Unknown

D04d. A person needs to take nPEP when one’s mucosa or damaged skin is exposed to the blood or other body fluids of people living with HIV.

[1] True [2] False [3] Unknown

D04e. A person needs to make sure they are not infected with HIV before taking nPEP.

[1] True [2] False [3] Unknown

D04f. A person needs to take nPEP even though condoms were used correctly throughout the process of sexual activities, regardless of the HIV status of sexual partners.

[1] True [2] False [3] Unknown

D04g. A person does not need to take nPEP if the source of exposure (e.g., sexual partners, peers of injection drug users, etc.) is not found to be infected with HIV.

[1] True [2] False [3] Unknown

D04h. The course of nPEP treatment is 28 days. Intermittent medication can be used as long as a cumulative use of nPEP lasts for 28 days.

[1] True [2] False [3] Unknown

D04i. Relevant tests are needed before, during and after taking nPEP.

[1] True [2] False [3] Unknown

D04j. NPEP can be taken within 72 hours after the occurrence of behaviours that may lead to the infection of HIV, and the sooner the better

[1] True [2] False [3] Unknown

D04k. When taking nPEP, there may be different degrees of drug reactions (e.g., dizziness, nausea, diarrhoea, fever, rash, liver and kidney damage) due to different personal physical conditions. NPEP users can stop taking nPEP independently according to their own drug reactions

[1] True [2] False [3] Unknown

D05. Have you ever used nPEP? [1] Yes [2] No

D06. If you had behaviors that may lead to the infection of HIV (e.g., unprotected sex and sharing needles), would you like to take nPEP?

[1] Yes (Jump to D07) [2] No (Jump to D08) [3] Not sure (Respond both D07 and D08)

D07. Which of the following factors can promote your use of nPEP? [Multiple choices]

[1] Unsafe behaviors [2] Sexual partners diagnosed with HIV [3] Sexual partners with unknown HIV status [4] Significant effectiveness [5] Convenient to access [6] Peer support [7] People you know have used nPEP [8] Other factors:

D08. What are the reasons that you do not want to use nPEP? [Multiple choices]

[1] Fear of privacy disclosure [2] Fear of social discrimination [3] Consistent condom use during sex behavior [4] Sexual partners without infection of HIV [5] fixed partner [6] Fear of side effects [7] High price [8] Difficulty to access [9] Have no awareness and trust of nPEP [10] Long course of treatment [11] Fear of influencing the treatment of other diseases [12] Other reasons:

D09. What is the maximum price of nPEP you can afford? (CNY=Chinese Yuan)

[1] <1000 CNY [2] 1000-3000 CNY [3] 3001-5000 CNY [4] >5000 CNY

D10. Where would you like to access nPEP？[Multiple choices, limited to 3]

[1] Hospital [2] CDC [3] CBOs [4] Pharmacy [5] Friends [6] Internet [7] Others:

D11. Which ways do you want to get information about nPEP? [Multiple choices, limited to 3]

[1] Internet/Social Media/APP [2] CBOs [3] CDC [4] Doctors [5] Friends

[6] Mass media (e.g., TV and Broadcast) [7] Newspapers and magazines [8] Training/ lectures [9] Others:

D12. What kinds of knowledge about nPEP do you want to acquire? [Multiple choices]

[1] Rationale of prevention [2] Applicable conditions [3] Regimen of nPEP [4] Course and time of nPEP [5] Locations to access nPEP [6] Price [7] Side effects [8] Others:

D13. Do you think whether nPEP should be promoted among drug users?

[1] Yes [2] No [3] Not sure

D14. If nPEP is promoted in China, do you think whether the condom use will change for yourself?

[1] Increased [2] Decreased [3] Unchanged

D15. If nPEP is promoted in China, do you think whether the condom uses will change among drug users?

[1] Increased [2] Decreased [3] Unchanged

D16. If nPEP is promoted in China, do you think whether the number of sexual partners will change for yourself?

[1] Increased [2] Decreased [3] Unchanged

D17. If nPEP is promoted in China, do you think whether the number of sexual partners will change among drug users?

[1] Increased [2] Decreased [3] Unchanged
