# Supplementary material for: Drug users’ awareness of and willingness to use HIV non-occupational post-exposure prophylaxis (nPEP) services in China: a mixed methods study
Source: BMC Infect Dis. 2022 Feb 14;22:151. doi: 10.1186/s12879-022-07106-x (PMC8842954; doi:10.1186/s12879-022-07106-x)
Supplement: Supplementary file 2 — Additional file 2. Variables and measures in quantitative study. [file 12879_2022_7106_MOESM2_ESM.docx]

**Additional file 2 Variables and Measures in quantitative study**

**1. Demographic information**

Demographic factors in this study included age (≤30/31-40/>40), sex (male/female), local household (yes/no), education level (senior high school and lower/college and above), monthly income (<1500 CNY/1500-3000 CNY/>3000 CNY) and marital status (currently unmarried/ currently married)

**2. AIDS knowledge**

The variable AIDS knowledge was measured using eight questions adopted from the National HIV Sentinel Surveillance in China with three responses “yes”, “no” and “not sure”. A correct answer for each question was given one point with a maximum of eight points and the “not sure” was treated as incorrect response. The variable was dichotomize based on the total score of the eight questions as “0-5” (i.e. poor knowledge) and “6-8” (i.e. good knowledge).

**3. Utilization of HIV prevention services**

Three questions were applied to query whether the participants had ever received condom promotion and distribution/ HIV counseling and testing services, community-based methadone maintenance treatment/ needle exchange program services, and peer education services in the past 12 months. The variable was categorized to binary with two categories “0-1” and “2-3” based on total number of HIV prevention services uptake.

**4. HIV-related high-risk behaviors**

The HIV-related high-risk behaviors included ever use multiple drugs (yes/no), drug use in the past 3 months (never /occasionally/more than once a month), alcohol use in the past 3 months (yes/no). HIV sexual behaviors were measured by two questions asking whether the participants had condomless sex after using drugs (yes/no) and whether they had condomless group sex after using drugs in the past year (yes/no).

**5. HIV risk perception**

A question was applied to query the participants perceived the risk of HIV infection among drug users in their living city with an option of answering “not serious or have no idea”, “moderate” and “serious”.

**6. Outcome variables**

For the outcome variable “awareness of nPEP”, the participants were asked whether they had heard of nPEP services before taking part in this study, with two responses “yes” and “no”. For the outcome variable “willingness to use nPEP”, the participants were asked whether they would use nPEP services if they were exposed to HIV in future, with two responses “yes” and “no”.
