# Supplementary material for: Drug users’ awareness of and willingness to use HIV non-occupational post-exposure prophylaxis (nPEP) services in China: a mixed methods study
Source: BMC Infect Dis. 2022 Feb 14;22:151. doi: 10.1186/s12879-022-07106-x (PMC8842954; doi:10.1186/s12879-022-07106-x)
Supplement: Supplementary file 3 — Additional file 3. Univariate analyses of the two outcome variables and sex-specific analyses. [file 12879_2022_7106_MOESM3_ESM.docx]

**Additional file 3 Univariate analyses of the two outcome variables and sex-specific analyses**

**Table S1. Factors associated with awareness of and willingness to use nPEP in univariate logistic regressions among drug users in China**

|  | **Having heard of nPEP** | | **Willing to use nPEP** | |
| --- | --- | --- | --- | --- |
| **Variables** | **Crude OR (95%CI)** | **P-value** | **Crude OR(95%CI)** | **P-value** |
| **Age (years)** |  |  |  |  |
| ≤30 | Ref |  | Ref |  |
| 31-40 | 0.270(0.151,0.485) | <0.001 | 0.521(0.305,0.890) | 0.017 |
| >40 | 0.172(0.098,0.301) | <0.001 | 0.309(0.190,0.503) | <0.001 |
| **Sex** |  |  |  |  |
| Male | Ref |  | Ref |  |
| Female | 0.191(0.112,0.327) | <0.001 | 0.598(0.395,0.907) | 0.016 |
| **Local household** |  |  |  |  |
| Yes | Ref |  | Ref |  |
| No | 3.052(1.911,4.876) | <0.001 | 1.887(1.177,3.025) | 0.008 |
| **Education level** |  |  |  |  |
| Senior high school and below | Ref |  | Ref |  |
| College and above | 3.971(2.516,6.269) | <0.001 | 1.653(1.079,2.534) | 0.021 |
| **Monthly income (CNY)** |  |  |  |  |
| <1500 | Ref |  | Ref |  |
| 1500-3000 | 0.953(0.500,1.817) | 0.883 | 2.730(1.502,4.962) | 0.001 |
| >3000 | 1.446(0.798,2.619) | 0.224 | 1.893(1.091,3.285) | 0.023 |
| **Marital status** |  |  |  |  |
| Currently unmarried | Ref |  | Ref |  |
| Currently married | 0.963(0.580,1.597) | 0.883 | 1.002(0.622,1.615) | 0.993 |
| **AIDS knowledge score** |  |  |  |  |
| 0-5 | Ref |  | Ref |  |
| 6-8 | 3.157(1.933,5.155) | <0.001 | 5.446(3.480,8.524) | <0.001 |
| **Utilization of HIV prevention services in the past year** |  |  |  |  |
| 0-1 | Ref |  | Ref |  |
| 2-3 | 1.139(0.681,1.905) | 0.621 | 1.494(0.931,2.399) | 0.097 |
| **Ever use multiple drugs** |  |  |  |  |
| No | Ref |  | Ref |  |
| Yes | 1.290(0.811,2.054) | 0.283 | 1.017(0.650,1.594) | 0.940 |
| **Drug use in the past 3 months** |  |  |  |  |
| Never | Ref |  | Ref |  |
| Occasionally | 1.765(0.999,3.118) | 0.051 | 0.545(0.332,0.897) | 0.017 |
| More than once a month | 3.560(2.060,6.154) | <0.001 | 1.136(0.684,1.887) | 0.621 |
| **Condomless sex after using drugs in the past year** |  |  |  |  |
| No | Ref |  | Ref |  |
| Yes | 2.545(1.634,3.963) | <0.001 | 1.934(1.256,2.980) | 0.003 |
| **Condomless sex with multiple partners after using drugs in the past year** |  |  |  |  |
| No | Ref |  | Ref |  |
| Yes | 2.213(1.237,3.960) | 0.007 | 2.527(1.326,4.814) | 0.005 |
| **Alcohol use in the past 3 months** |  |  |  |  |
| No | Ref |  | Ref |  |
| Yes | 0.658(0.421,1.029) | 0.066 | 0.274(0.169,0.443) | <0.001 |
| **HIV risk perception** |  |  |  |  |
| Not serious or have no idea | Ref |  | Ref |  |
| Moderate | 4.732(2.852,7.852) | <0.001 | 5.038(2.861,8.870) | <0.001 |
| Serious | 2.196(1.196,4.032) | 0.011 | 0.605(0.344,1.064) | 0.081 |
| **Having heard of nPEP** |  |  |  |  |
| No | NA | NA | Ref |  |
| Yes | NA | NA | 5.692(3.320,9.760) | <0.001 |
| Note: nPEP, non-occupational post-exposure prophylaxis; CNY, Chinese Yuan (1 CNY=0.14 USD); NA, not applicable; AOR, adjusted odds ratio; CI, confidence interval | | | | |

**Table S2. Factors associated with awareness of and willingness to use nPEP in univariate logistic regressions among male drug users in China**

|  | **Having heard of nPEP** | | **Willing to use nPEP** | |
| --- | --- | --- | --- | --- |
| **Variables** | **Crude OR (95%CI)** | **P-value** | **Crude OR(95%CI)** | **P-value** |
| **Age (years)** |  |  |  |  |
| ≤30 | Ref |  | Ref |  |
| 31-40 | 0.420(0.213,0.829) | 0.012 | 0.521(0.258,1.052) | 0.069 |
| >40 | 0.137(0.065,0.289) | <0.001 | 0.285(0.148,0.546) | <0.001 |
| **Local household** |  |  |  |  |
| Yes | Ref |  | Ref |  |
| No | 2.160(1.257,3.712) | 0.005 | 1.465(0.835,2.572) | 0.183 |
| **Education level** |  |  |  |  |
| Senior high school and below | Ref |  | Ref |  |
| College and above | 3.060(1.767,5.301) | <0.001 | 2.150(1.230,3.758) | 0.007 |
| **Monthly income (CNY)** |  |  |  |  |
| <1500 | Ref |  | Ref |  |
| 1500-3000 | 1.225(0.567,2.647) | 0.606 | 2.512(1.134,5.568) | 0.023 |
| >3000 | 1.323(0.655,2.671) | 0.435 | 3.309(1.594,6.870) | 0.001 |
| **Marital status** |  |  |  |  |
| Currently unmarried | Ref |  | Ref |  |
| Currently married | 1.060(0.579,1.939) | 0.851 | 1.323(0.695,2.518) | 0.394 |
| **AIDS knowledge score** |  |  |  |  |
| 0-5 | Ref |  | Ref |  |
| 6-8 | 3.198(1.774,5.767) | <0.001 | 4.349(2.423,7.805) | <0.001 |
| **Utilization of HIV prevention services in the past year** |  |  |  |  |
| 0-1 | Ref |  | Ref |  |
| 2-3 | 1.646(0.880,3.078) | 0.119 | 2.920(1.558,5.474) | 0.001 |
| **Ever use multiple drugs** |  |  |  |  |
| No | Ref |  | Ref |  |
| Yes | 0.974(0.562,1.688) | 0.926 | 1.014(0.572,1.797) | 0.963 |
| **Drug use in the past 3 months** |  |  |  |  |
| Never | Ref |  | Ref |  |
| Occasionally | 1.725(0.863,3.447) | 0.123 | 0.989(0.497,1.970) | 0.975 |
| More than once a month | 2.580(1.317,5.058) | 0.006 | 1.204(0.612,2.372) | 0.591 |
| **Condomless sex after using drugs in the past year** |  |  |  |  |
| No | Ref |  | Ref |  |
| Yes | 1.814(1.073,3.067) | 0.026 | 1.485(0.865,2.550) | 0.152 |
| **Condomless sex with multiple partners after using drugs in the past year** |  |  |  |  |
| No | Ref |  | Ref |  |
| Yes | 1.474(0.763,2.848) | 0.249 | 1.597(0.783,3.256) | 0.198 |
| **Alcohol use in the past 3 months** |  |  |  |  |
| No | Ref |  | Ref |  |
| Yes | 0.785(0.456,1.350) | 0.381 | 0.646(0.361,1.157) | 0.141 |
| **HIV risk perception** |  |  |  |  |
| Not serious or have no idea | Ref |  | Ref |  |
| Moderate | 3.786(2.068,6.929) | <0.001 | 5.286(2.638,10.592) | <0.001 |
| Serious | 2.183(1.034,4.609) | 0.041 | 0.739(0.352,1.551) | 0.424 |
| **Having heard of nPEP** |  |  |  |  |
| No | NA | NA | Ref |  |
| Yes | NA | NA | 4.310(2.345,7.921) | <0.001 |
| Note: nPEP, non-occupational post-exposure prophylaxis; CNY, Chinese Yuan (1 CNY=0.14 USD); NA, not applicable; AOR, adjusted odds ratio; CI, confidence interval | | | | |

**Table S3. Factors associated with awareness of nPEP in multivariate logistic regressions among male drug users in China**

| **Variables** | **β** | **SE** | **AOR(95%CI)** | **P-value** |
| --- | --- | --- | --- | --- |
| **Age (years)** |  |  |  |  |
| ≤30 | Ref |  | 1 |  |
| 31-40 | -0.756 | 0.381 | 0.465(0.221,0.981) | 0.044 |
| >40 | -2.989 | 0.434 | 0.137(0.059,0.320) | <0.001 |
| **AIDS knowledge score** |  |  |  |  |
| 0-5 | Ref |  | 1 |  |
| 6-8 | 0.844 | 0.340 | 2.325(1.193,4.531) | 0.013 |
| **HIV risk perception** |  |  |  |  |
| Not serious or have no idea | Ref |  | 1 |  |
| Moderate | 0.800 | 0.350 | 2.226(1.120,4.424) | 0.022 |
| Serious | 0.775 | 0.457 | 2.170(0.887,5.310) | 0.090 |
| Note: nPEP, non-occupational post-exposure prophylaxis; CNY, Chinese Yuan (1 CNY=0.14 USD); NA, not applicable; AOR, adjusted odds ratio; CI, confidence interval | | | | |

**Table S4. Factors associated with willingness to use nPEP in multivariate logistic regressions among male drug users in China**

| **Variables** | **β** | **SE** | **AOR(95%CI)** | **P-value** |
| --- | --- | --- | --- | --- |
| **Monthly income (CNY)** |  |  |  |  |
| <1500 | Ref |  | 1 |  |
| 1500-3000 | 1.125 | 0.502 | 3.080(1.151,8.245) | 0.025 |
| >3000 | 1.456 | 0.476 | 4.287(1.687,10.896) | 0.002 |
| **AIDS knowledge score** |  |  |  |  |
| 0-5 | Ref |  | 1 |  |
| 6-8 | 1.505 | 0.387 | 4.506(2.110,9.624) | <0.001 |
| **HIV risk perception** |  |  |  |  |
| Not serious or have no idea | Ref |  | 1 |  |
| Moderate | 0.900 | 0.410 | 2.460(1.103,5.491) | 0.028 |
| Serious | -0.796 | 0.479 | 0.451(0.176,1.154) | 0.097 |
| **Utilization of HIV prevention services in the past year** |  |  |  |  |
| 0-1 | Ref |  | 1 |  |
| 2-3 | 0.894 | 0.383 | 2.446(1.153,5.186) | 0.020 |
| **Having heard of nPEP** |  |  |  |  |
| No | Ref |  | 1 |  |
| Yes | 1.104 | 0.368 | 3.016(1.465,6.207) | 0.003 |
| Note: nPEP, non-occupational post-exposure prophylaxis; CNY, Chinese Yuan (1 CNY=0.14 USD); NA, not applicable; AOR, adjusted odds ratio; CI, confidence interval | | | | |

**Table S5. Factors associated with awareness of and willingness to use nPEP in univariate logistic regressions among female drug users in China**

|  | **Having heard of nPEP** | | **Willing to use nPEP** | |
| --- | --- | --- | --- | --- |
| **Variables** | **Crude OR (95%CI)** | **P-value** | **Crude OR(95%CI)** | **P-value** |
| **Age (years)** |  |  |  |  |
| ≤30 | Ref |  | Ref |  |
| 31-40 | 0.082(0.010,0.682) | 0.021 | 0.688(0.284,1.665) | 0.407 |
| >40 | 0.522(0.193,1.414) | 0.201 | 0.442(0.198,0.985) | 0.046 |
| **Local household** |  |  |  |  |
| Yes | Ref |  | Ref |  |
| No | 2.083(0.615,7.053) | 0.238 | 2.481(0.890,6.919) | 0.082 |
| **Education level** |  |  |  |  |
| Senior high school and below | Ref |  | Ref |  |
| College and above | 3.182(1.198,8.454) | 0.020 | 0.827(0.383,1.786) | 0.628 |
| **Monthly income (CNY)** |  |  |  |  |
| <1500 | Ref |  | Ref |  |
| 1500-3000 | 0.655(0.137,3.129) | 0.596 | 2.923(1.180,7.243) | 0.021 |
| >3000 | 1.728(0.440,6.792) | 0.433 | 0.762(0.315,1.841) | 0.545 |
| **Marital status** |  |  |  |  |
| Currently unmarried | Ref |  | Ref |  |
| Currently married | 0.574(0.157,2.089) | 0.399 | 0.699(0.326,1.500) | 0.358 |
| **AIDS knowledge score** |  |  |  |  |
| 0-5 | Ref |  | Ref |  |
| 6-8 | 2.198(0.790,6.113) | 0.131 | 6.625(3.246,13.522) | <0.001 |
| **Utilization of HIV prevention services in the past year** |  |  |  |  |
| 0-1 | Ref |  | Ref |  |
| 2-3 | 0.468(0.169,1.291) | 0.142 | 0.592(0.279,1.257) | 0.173 |
| **Ever use multiple drugs** |  |  |  |  |
| No | Ref |  | Ref |  |
| Yes | 1.626(0.574,4.600) | 0.360 | 0.904(0.421,1.943) | 0.796 |
| **Drug use in the past 3 months** |  |  |  |  |
| Never | Ref |  | Ref |  |
| Occasionally | 0.468(0.095,2.312) | 0.351 | 0.165(0.067,0.407) | <0.001 |
| More than once a month | 3.042(1.030,8.984) | 0.044 | 0.997(0.428,2.318) | 0.994 |
| **Condomless sex after using drugs in the past year** |  |  |  |  |
| No | Ref |  | Ref |  |
| Yes | 1.533(0.510,4.609) | 0.447 | 2.436(1.053,5.636) | 0.037 |
| **Condomless sex with multiple partners after using drugs in the past year** |  |  |  |  |
| No | Ref |  | Ref |  |
| Yes | 2.912(0.704,12.044) | 0.140 | 11.692(1.458,93.790) | 0.021 |
| **Alcohol use in the past 3 months** |  |  |  |  |
| No | Ref |  | Ref |  |
| Yes | 0.390(0.151,1.010) | 0.052 | 0.045(0.015,0.137) | <0.001 |
| **HIV risk perception** |  |  |  |  |
| Not serious or have no idea | Ref |  | Ref |  |
| Moderate | 2.475(0.768,7.978) | 0.129 | 3.820(1.317,11.081) | 0.014 |
| Serious | 1.485(0.435,5.072) | 0.528 | 0.424(0.171,1.052) | 0.064 |
| **Hearing of nPEP ^a^** |  |  |  |  |
| No | NA |  | Ref |  |
| Yes | NA | NA | 24.000(3.112,185.082) | 0.002 |
| Note: nPEP, non-occupational post-exposure prophylaxis; CNY, Chinese Yuan (1 CNY=0.14 USD); NA, not applicable; AOR, adjusted odds ratio; CI, confidence interval  ^a^ There was only one female participant reported no willingness to use nPEP among those who had ever heard of nPEP due to small sample size in sex-specific analysis. The phenomenon of data imbalance can lead to large deviation of the maximum likelihood estimate. The variable “hearing of nPEP” was not included in multivariate logistic regressions targeting nPEP willingness. | | | | |

**Table S6. Factors associated with awareness of nPEP in multivariate logistic regressions among female drug users in China**

| **Variables** | **β** | **SE** | **AOR(95%CI)** | **P-value** |
| --- | --- | --- | --- | --- |
| **Education level** |  |  |  |  |
| Senior high school and below | Ref |  | 1 |  |
| College and above | 1.844 | 0.603 | 6.319(1.939,20.594) | 0.002 |
| **Alcohol use in the past 3 months** |  |  |  |  |
| No | Ref |  | 1 |  |
| Yes | -1.400 | 0.607 | 0.247(0.075,0.811) | 0.021 |
| Note: nPEP, non-occupational post-exposure prophylaxis; CNY, Chinese Yuan (1 CNY=0.14 USD); NA, not applicable; AOR, adjusted odds ratio; CI, confidence interval | | | | |

**Table S7. Factors associated with willingness to use nPEP in multivariate logistic regressions among female drug users in China**

| **Variables** | **β** | **SE** | **AOR(95%CI)** | **P-value** |
| --- | --- | --- | --- | --- |
| **Local household** |  |  |  |  |
| Yes | Ref |  | 1 |  |
| No | 2.104 | 0.710 | 8.201(2.039,32.988) | 0.003 |
| **AIDS knowledge score** |  |  |  |  |
| 0-5 | Ref |  | 1 |  |
| 6-8 | 2.174 | 0.549 | 8.792(2.998,25.787) | <0.001 |
| **Condomless sex after using drugs in the past year** |  |  |  |  |
| No | Ref |  | 1 |  |
| Yes | 1.948 | 0.592 | 7.016(2.198,22.395) | 0.001 |
| **Alcohol use in the past 3 months** |  |  |  |  |
| No | Ref |  | 1 |  |
| Yes | -3.431 | 0.702 | 0.032(0.008,0.128) | <0.001 |
| Note: nPEP, non-occupational post-exposure prophylaxis; CNY, Chinese Yuan (1 CNY=0.14 USD); NA, not applicable; AOR, adjusted odds ratio; CI, confidence interval | | | | |
