# Supplementary material for: Drug users’ awareness of and willingness to use HIV non-occupational post-exposure prophylaxis (nPEP) services in China: a mixed methods study
Source: BMC Infect Dis. 2022 Feb 14;22:151. doi: 10.1186/s12879-022-07106-x (PMC8842954; doi:10.1186/s12879-022-07106-x)
Supplement: Supplementary file 4 — Additional file 4. Suggestions for nPEP service in qualitative study. [file 12879_2022_7106_MOESM4_ESM.docx]

**Additional file 4 Suggestions for nPEP service in qualitative study**

Most (15/19) participants thought it was necessary to routinize nPEP services among drug users. However, two participants did not support nPEP routinization because they thought drug users never mind their own health.

*I definitely think the routinization of nPEP is necessary. At least the service will help me to prevent HIV infection ahead of time. I did not know the service in detail before. (27-year-old, QD09)*

*There is no need to routinize (nPEP). Drug users care nothing if they have taken drugs. Because they were consciously aware that taking drugs is by no means a right thing, still they do so. They are indifferent to other things. (23-year-old, QD06)*

For those who supported the routinization of nPEP, the most popular propaganda was via social media (e.g. WeChat), followed by face-to-face communications with healthcare staff or local CBOs. Other propaganda form included posters, bill boards and lectures.

*You can promote this service (nPEP) through WeChat. But you would better not to establish WeChat group. You can give a concise introduction about nPEP through Internet, and then let them (drug users) promote it (nPEP) immediately. (24-year-old, QD05)*

As for the institutions providing nPEP services, 10 (52.6%) participants preferred pharmacies for its convenience and superiority of privacy protection. Four (21.1%) participants chose hospitals due to authoritativeness. In addition, there was a participant suggesting to provide nPEP services through Internet.

*It is undoubtedly that pharmacies are suitable to provide nPEP service for its convenience and security. It is also acceptable that CDC provide this service. By comparison, it is under high risk to get nPEP medications from acquaintance or friends. (50-year-old, SH03)*

In Shanghai, only two (20%) participants could afford nPEP medications. By contrast, six (66.7%) participants could afford this service in Qingdao who indicated that they attached more importance to the effect than the cost. Most participants thought current price of nPEP medications is a little high and the affordable price should be no more than 2000 CNY. There was a participant suggesting that the cost of nPEP should be partly reimbursed by the government.

*If possible, I think the government should share half or a portion of the (nPEP medication) cost. Then we bear the rest. (30-year-old, QD03)*
